# *De novo* Transcriptome Sequencing of MeJA-Induced *Taraxacum koksaghyz* Rodin to Identify Genes Related to Rubber Formation

**DOI:** 10.1038/s41598-017-14890-z

**Published:** 2017-11-16

**Authors:** XinWen Cao, Jie Yan, JiLiang Lei, Jin Li, JianBo Zhu, HuiYan Zhang

**Affiliations:** College of Life Science, University of Shi He Zi, Xin Jiang, 832000 China

## Abstract

Increase in the consumption of natural rubber (NR) has necessitated the identification of alternative sources of NR. The quality of NR produced by *Taraxacum koksaghyz* Rodin (TKS) is comparable to that from *Hevea brasiliensis* (*H.brasiliensis*), and therefore, TKS is being considered as an alternative source of NR. Here, we sequenced the TKS root transcriptome after wild TKS seedlings were treated with methyl jasmonate (MeJA) for 0, 6, and 24 h. The clean reads generated for each experimental line were assembled into 127,833 unigenes. The Kyoto encyclopedia of genes and genomes pathway prediction suggested that methyl jasmonate regulated secondary metabolism in TKS. Differential expression analysis showed that the expression of *HMGCR*, *FPPS*, *IDI*, *GGPPS*, and *REF/SRPP* increased with methyl jasmonate treatment. Interestingly, differential expression analysis of the jasmonate (JA)-related transcription factors (TFs), indicated that certain genes encoding these transcription factors (namely, bHLH, MYB, AP2/EREBP, and WRKY) showed the same expression pattern in the lines treated for 6 h and 24 h. Moreover, *HMGCR* was up-regulated in the transgenic seedlings overexpressing *DREB*. We predicted that methyl jasmonate regulated secondary metabolism and affected rubber biosynthesis via the interaction of the JA-related TFs with genes associated with rubber biosynthesis in TKS.

## Introduction

Natural rubber (NR) (cis-1,4-polyisoprene) is an important industrial material, which plays a pivotal role in the production of industrial and medical products, especially in the military-industrial complex. The demand for rubber has increased with the rapid economic development of recent years. In 2010, the rubber consumption of the world was approximately 10 million metric tons and the price of NR had increased 7-fold compared to that in 1997^1^; in 2014, the global rubber consumption was estimated to be 12 million tons^2^. *Hevea brasiliensis*, a tropical/subtropical tree, is the primary source of NR. Unfortunately, *H. brasiliensis* alone cannot meet the increasing demand for NR owing to the limitations of its cultivation area and a variety of biotic and abiotic stresses. Currently, the tree suffers severely from attack by various plant diseases and insect pests such as South American leaf blight (SALB, *Microcyclus ulei*), abnormal leaf fall (*Phytopthora* spp.), powdery mildew (*Oidium heveae*), Corneyspora leaf fall (*Cornyespora cassiicola*), pink disease (*Corticium salmonicolor*), and white root disease (*Rigidoporus* spp.) Among these, SALB caused by *Microcyclus ulei* is the most serious threat to the NR industry worldwide as it has negatively impacted the growth of *H. brasiliensis* in Central and South America^3^. At present, the world supply of NR is highly dependent on the Southeast Asian plantations^3^; however, SALB is disseminated by human activities as well as by natural spore dispersal of the pathogen, and might eventually reach the Asian rubber tree populations^4^. In addition, *H. brasiliensis* is facing a serious menace of tapping panel dryness (TPD), and annual loss in rubber production due to TPD accounted for 10–40% with an incidence of 12–50%^5^. Since no effective treatments for TPD exist^6,7^, TPD causes substantial losses in NR production. According to the economic model prediction, NR production will not be sufficient by 2020^8^. Therefore, alternative sources of NR are urgently required.

*Taraxacum koksaghyz* Rodin (TKS), commonly known as the Russian dandelion, is a perennial, diploid species that contains significant amount of rubber, especially in the roots (about 24% on a dry weight basis in improved TKS). The property of rubber from TKS is comparable to that from *H. brasiliensis* ^9^. TKS was industrially cultivated in the 1930–1940 s to develop it into a viable source of NR by limiting access to rubber tree plantations^10^. Because of its strong adaptability, short growth cycle (wild TKS can accumulate 10% rubber in 1 cultivation year),^11^ and the ability to be cultivated on a large-scale with higher survival rate, TKS has received widespread attention in recent years^12^ and is being developed as an alternative source of commercial grade NR^13,14^. Unfortunately, TKS possesses limited rubber content. To improve rubber production of TKS, numerous studies have aimed to elucidate the mechanism of rubber biosynthesis^15–19^. The biosynthetic pathway of NR has now been largely elucidated; the terpenoid metabolism of plants depends on the mevalonate (MVA) and non-mevalonate (DOXP) pathways, which also function in rubber biosynthesis^20,21^. Particularly, the MVA pathway plays a key role, and the major enzymes involved have been identified, namely HMGCR, HMGS, IDI, FPPS, and GGPPS^22–24^. However, assessment of the role of individual genes in rubber biosynthesis is not sufficient to improve the quality of rubber. Furthermore, the detailed mechanism of rubber biosynthesis in TKS is still unclear.

Hormones play an important role in regulating plant growth and development, including seed germination, root elongation, bud dormancy, and secondary metabolism^25–29^. Recent evidence showed that the application of exogenous hormones promoted the yield of plant secondary metabolites^30^. Exogenously applied plant growth regulators, such as the combination of Vitamin C, Vitamin E, methyl jasmonate (MeJA), and bromide (Br) enhanced the yield of rice at high temperature due to enhanced photosynthesis, spikelet fertility, and grain filling^31^. Ethephon (2-chloroethylphosphonic acid) has been used as an effective stimulant to prolong the latex flow in rubber plants, which resulted in higher yields of rubber^32^. Jasmonates, including jasmonic acid (JA) and its methyl ester (MeJA), amino acid conjugates, and other metabolites such as 12-OH-JA^33^, also play a central role in regulating the biosynthesis of many secondary metabolites^31^. Although efforts for understanding the role of hormones in plant metabolism have been implemented, the detailed mechanism remains unclear. Currently, high-throughput sequencing technologies are being used to address this question. Investigation of transcriptomes provided evidence about the role of jasmonates in inducing secondary metabolism. Analysis of MeJA-induced transcriptomes in *Polygonum minus* ^34^, *Panax quinquefolius* ^35^, *Centella asiatica* ^36^, and *Salvia miltiorrhiza* Bunge^37^ demonstrated that genes involved in secondary metabolism were up-regulated. In addition, application of 0.5% (w/w) MeJA in lanolin paste on tomatoes in the mature green (MG) stage resulted in lycopene accumulation^38^. A synthetic approach that combines the application of exogenous hormones to elicit plant secondary metabolism and subsequent transcriptome analysis would further the research in TKS. Here, we applied exogenous MeJA to stimulate the secondary metabolism of TKS at a developmental stage. Sequencing of the transcriptome was performed after 0 h, 6 h and 24 h of treatment; the resulting datasets would provide information about the rubber biosynthetic pathway as well as the mechanisms of MeJA-modulated growth and development of TKS. Furthermore, this study would promote further research for enhancing rubber productivity in TKS.

## Results and Discussion

### Transcriptome sequencing and *de novo* assembly

The wild TKS seedlings were treated with 0.8 mmol/L MeJA, and the roots were harvested from the treated lines (0, 6, and 24 h treatment). Transcriptome sequencing was performed to identify the MeJA-responsive genes involved in modulating rubber biosynthesis in TKS. Nine sequencing cDNA libraries were constructed (including three replicates in each treated line). Approximately 44,703,137, 43,834,502, and 45,558,831 clean average reads were generated for each experimental line after filtering and removing the adapters, the low quality reads, and unknown nucleotides. The number of clean reads obtained in the cDNA library of each experimental line confirmed that the length of the generated transcripts and the abundance of the detected genes were sufficient. The percentage of clean reads was more than 90%, and the Q20 percentage (sequencing error rate < 1%) and GC content were 95% and 44%, respectively among all libraries. These parameters indicated that the transcriptome library was of high quality. Further, 44,703,137, 43,834,502, and 45,558,831 clean reads were assembled de novo using Trinity, which revealed 226,524 transcripts with mean length of 857 nucleotides (nt) and N50 length of 1,413 bp. These represent 127,833 unigenes and would function as reference sequences for further analysis (Table 1).

**Table 1:** Assembly and annotation statistics of transcriptomes.

Clean reads	
MJ0_R1	45,012,356
MJ0_R2	46,976,300
MJ0_R3	42,120,756
MJ6_R1	41,091,182
MJ6_R2	42,796,854
MJ6_R3	47,615,470
MJ24_R1	46,811,300
MJ24_R2	43,596,792
MJ24_R3	46,268,400
**Trinity Assembly**	
Number of transcripts	226,524
Mean length of transcripts	857
N50 of transcripts	1413
Number of unigenes	127,833
Mean length of unigenes	638
N50 of unigenes	1081
**Annotation**	
NR	54,508
NT	37,653
KO	22,904
SwissProt	48,114
PFAM	44,372
GO	45,232
KOG	27,321
Annotated in at least one Database	74,997

MJ0, MJ6, and MJ24 indicate the seedlings treated by MeJA for 0 h, 6 h, and 24 h; R1, R2, and R3 indicate the three biological replicates of each treated line.

### Function annotation analysis

For function annotation analysis, 127,833 unigenes were predicted by the nucleotide collection (NR/NT), Pfam, and Swiss-Prot databases. Among the products of the predicted genes, 42.64% of the putative proteins showed similarity to sequences in the non-redundant protein database (NR) of NCBI, and 29.45%, 17.91%, 37.63%, 34.71%, 35.38%, and 21.37% of the genes showed functional annotation in the NT, Kyoto Encyclopedia of Genes and Genomes (KEGG) orthology (KO), Swiss-Prot, protein families (PFAM), gene ontology (GO), and eukaryotic orthologous groups (KOG) databases. Furthermore, 58.66% of these genes showed hits in at least one database (Table 1). Particularly, the predicted result of the NR database revealed that the matched species were *Vitis vinifera*, *Sesamum indicum*, *Coffea canephora*, *Citrus sinensis*, and *Nicitiana sylvestris*, which accounted for 7.9%, 4.6%, 4.2%, 3.7%, and 3.3% of the unigenes, respectively. However, the majority showed no matches with any known species, indicating that complete information about TKS is not available (Supplementary Fig. S1). To assess the annotation quality, the similarity distribution of NR database annotation was calculated. Eighty-three percent showed more than 60% similarity, with E-value ≤ 1e^−5^ (Supplementary Figs S2, S3).

**Figure 1:**
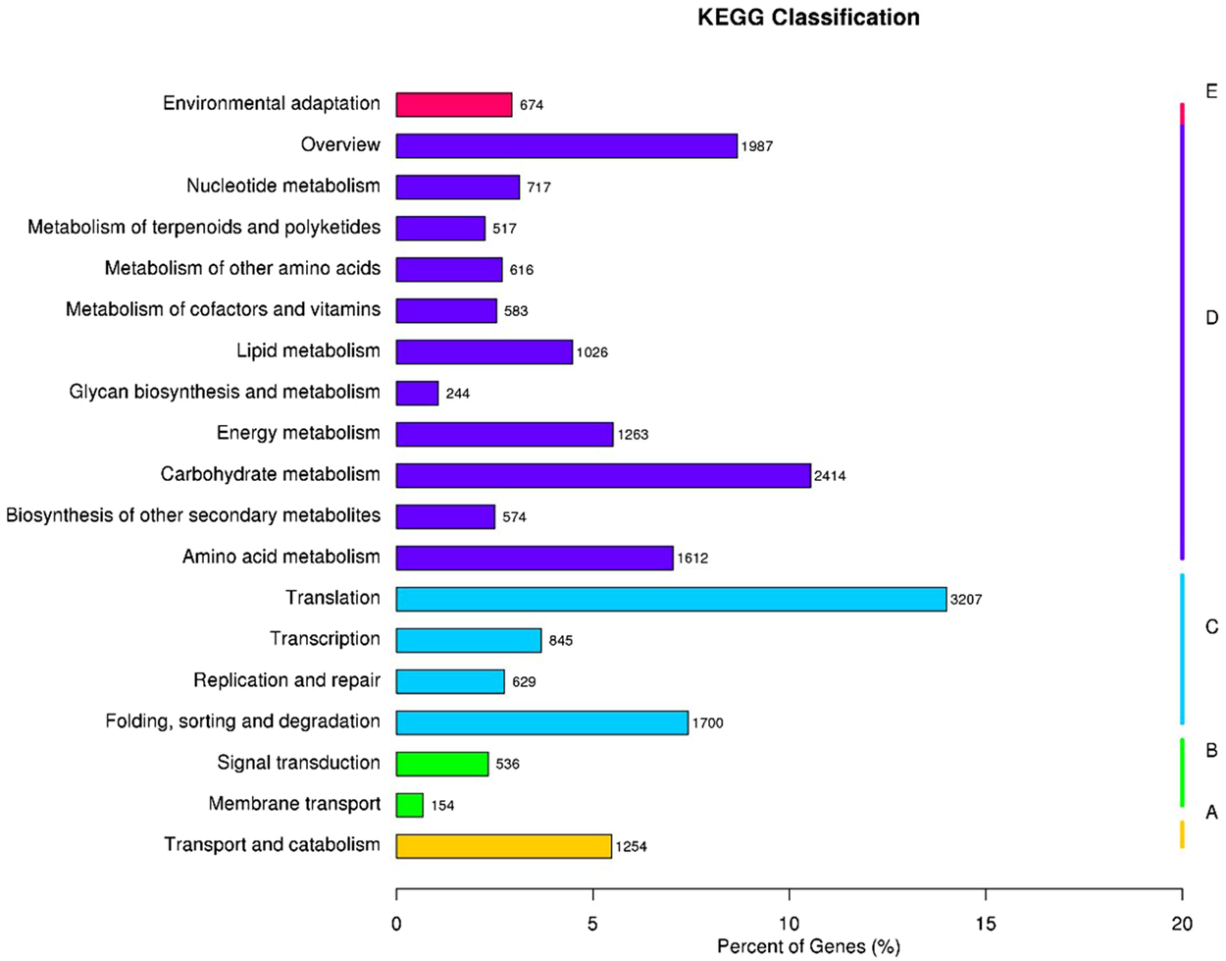
KEGG (Kyoto Encyclopedia of Genes and Genomes) pathway classification of the transcriptomes. The x-axis shows the gene numbers and percentages for each different term.

GO annotation revealed that the most predicted peptides were categorized into cellular process, metabolic process, and the single-organism process of biological process categories. In addition, the most represented molecular function was binding and catalytic activity, whereas the majority of the genes belonged to the cell and cellular component categories (Supplementary Fig. S4). We obtained a general idea of KOG category based on homology-detection. All genes were divided into twenty-six categories. General function prediction was distinct of all those, followed by those involved in post-translational modification, protein turnover, chaperone function, translation, ribosomal structure, and biogenesis (Supplementary Fig. S5). The KEGG pathway prediction illustrates the involvement of genes in various cellular pathways; The majority of the the genes (3,207) were assigned to translation, carbohydrate metabolism (2,414), terpenoid and polyketide metabolism (517), and biosynthesis of other secondary metabolites (574) (Fig. 1). Interestingly, the majority of the genes that responded to MeJA were involved in metabolism.

### Identification of differentially expressed genes

**Figure 2:**
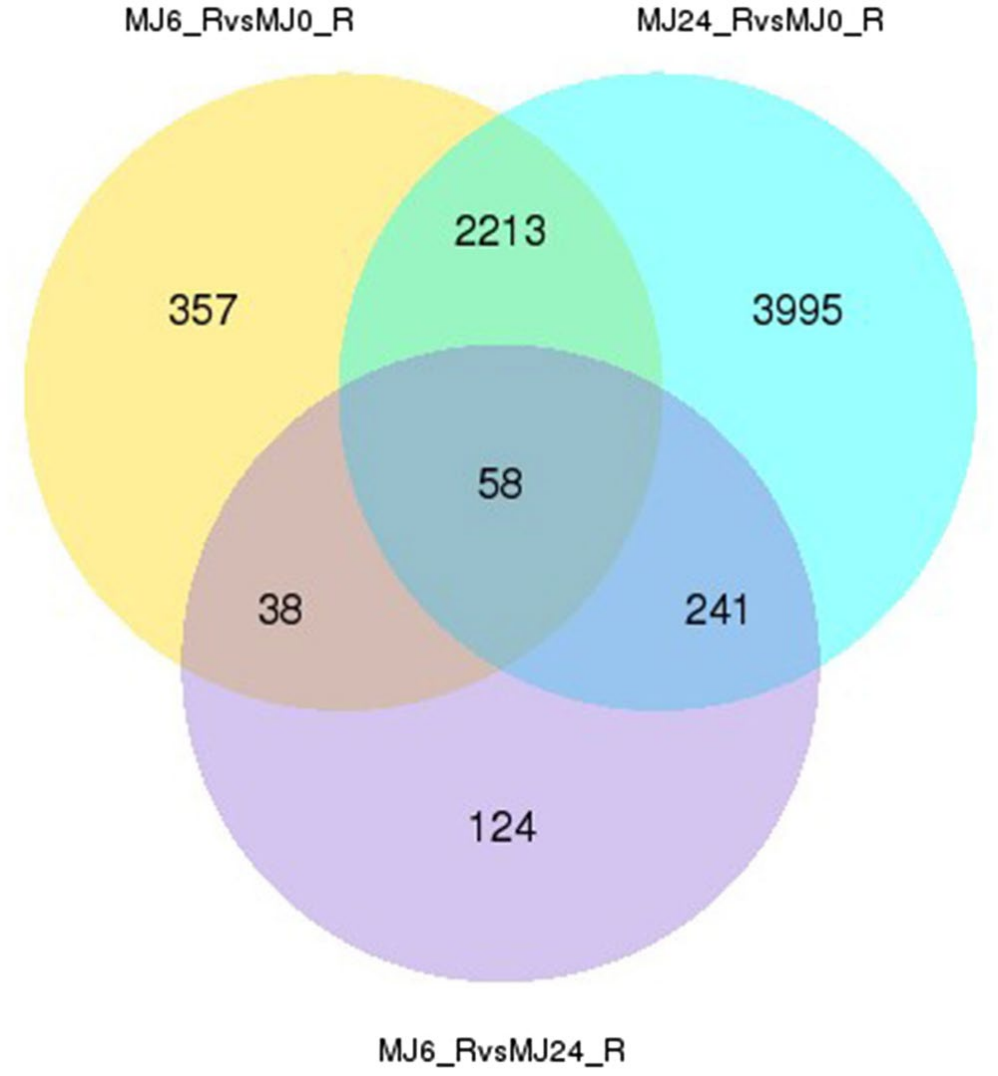
DEGs comparative statistics. Venn diagram illustrating the shared and unique DEG transcripts from the comparative analysis between different groups (group 1:MJ6-vs-MJ0, group 2: MJ24-vs-MJ0, group 3: MJ6-vs-MJ24); The numbers in each circle indicate the number of DEGs for the corresponding group, and the overlaps indicate the number of shared DEGs between different groups; MJ0, MJ6, and MJ24 indicate the lines treated with MeJA for 0 h, 6 h, and 24 h, respectively.

**Figure 3:**
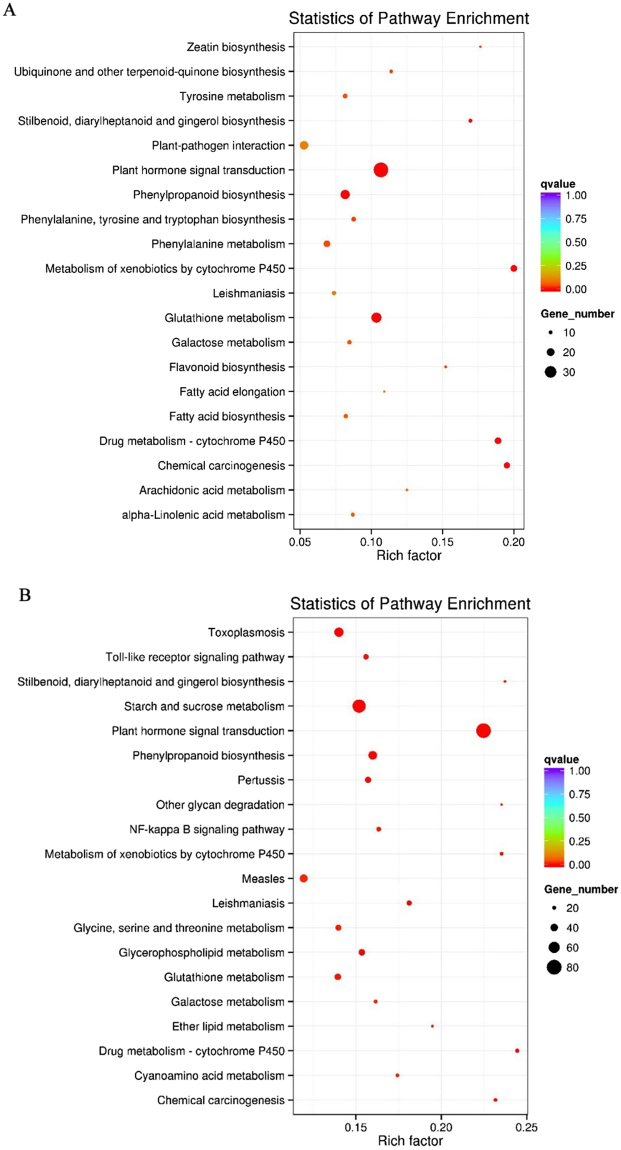
Statistics of DEGs KEGG pathway enrichment for the MJ0-vs-MJ6 (**A**) and MJ0-vs-MJ24 (**B**) groups. Distribution of the differentially expressed genes obtained from the comparative analysis between MJ0 and MJ6 (A), MJ0 and MJ24 (B); MJ0, MJ6, and MJ24 indicate the lines treated with MeJA for 0 h, 6 h and 24 h. The Y-axis on the right side represents the number of genes.

Next, we analyzed the differential gene expression pattern. A total of 9,634 statistically significant differentially expressed genes (DEGs) were identified based on the criteria of Padj <0.05 and |log_2_ Fold change| > 1. Of these, 2,666 DEGs were obtained between the 0 h and the 6 h-treated lines (1,842 up-regulated and 824 down-regulated) and 6,507 DEGs were obtained between the 0 h and the 24 h-treated lines (3,707 up-regulated and 2,800 down-regulated) (Supplementary Fig. S6A,B). Comparison of the transcriptomes of the 0 h, 6 h, and 24 h-treated lines revealed that only 124 DEGs belonged to the 6 h versus 24 h-treated lines, suggesting that the pattern of gene expression was concordant in the 6 h and 24 h-treated samples (Fig. 2). GO and KEGG enrichment analyses were conducted to further explore the possible role of these DEGs. Comparison of the 0 h and the 6 h-treated lines showed that the GO terms are mainly associated with catalytic, transferase, and oxidoreductase activities. Similarly, the molecular function of the remaining DEGs was related to metal ion binding and cation binding in the 0 h versus 24 h-treated group (Supplementary Fig. S7A,B). Interestingly, the KEGG analysis results of DEGs annotation for both the 0 h versus 6 h-treated and the 0 h versus 24 h-treated lines showed that the KEGG pathways were associated with plant hormone signal transduction, phenylpropanoid biosynthesis, glutathione metabolism, drug metabolism-cytochrome P450, metabolism of xenobiotics by cytochrome P450, and alpha-linolenic acid metabolism. All the pathways, except those for alpha-linolenic acid metabolism, metabolism of xenobiotics by cytochrome P450, and plant hormone signal transduction, are involved in the biosynthesis of secondary metabolites (Fig. 3), indicating that MeJA regulates secondary metabolism in TKS.

### Expression of α-linolenic acid metabolism-specific and jasmonate signaling pathway-specific genes

**Figure 4:**
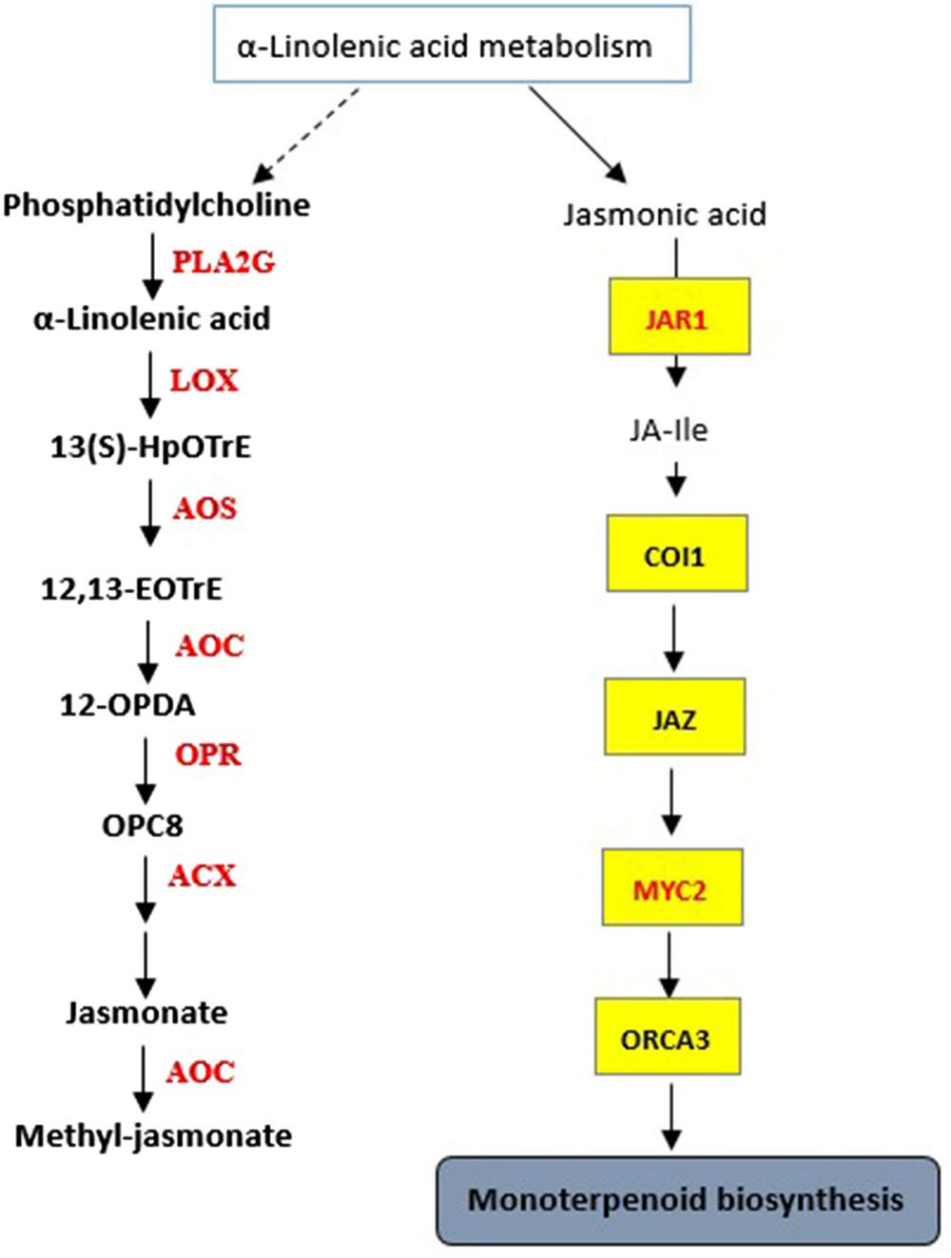
The expression pattern analysis of genes involved in JA biosynthesis and α-linolenic acid metabolic pathway. Red indicates up-regulation of gene expression. PLA2G, secretory phospholipase A2; LOX, lipoxygenase; AOS, allene oxide synthase; AOC, allene oxide cyclase; OPR, 12-oxophytodienoic acid reductase; ACX, acyl-CoA oxidase; JAR1, jasmonic acid-amino synthetase; COI-1, coronatine-insensitive protein 1; JAZ, jasmonate ZIM domain-containing protein; transcription factor MYC2; ORCA3, AP2-domain DNA-binding protein ORCA3.

**Figure 5:**
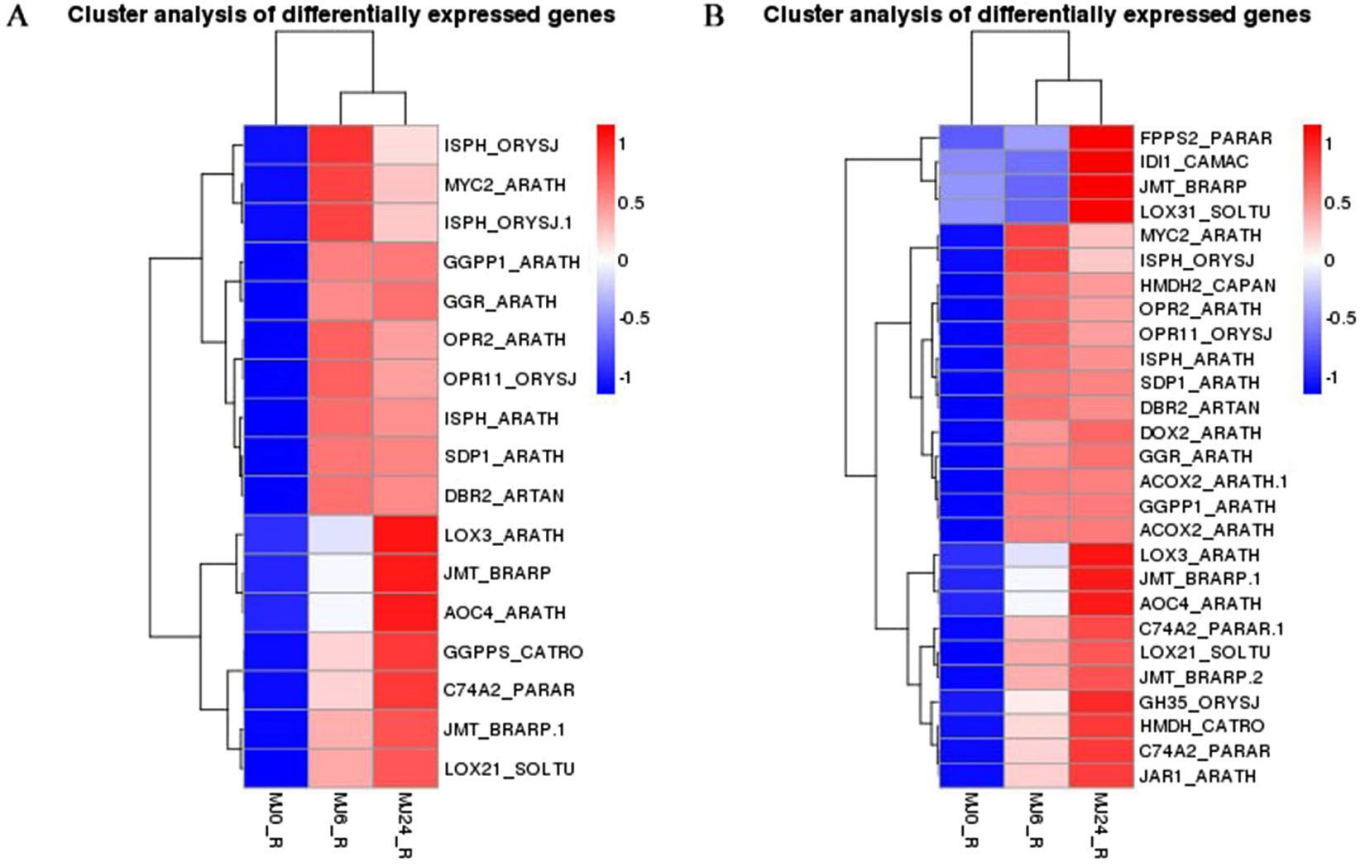
Heat map of the differentially expressed genes in the α-linolenic acid metabolic pathway and the rubber biosynthesis pathway. MJ0, MJ6, and MJ24 indicate the experiment lines treated by MeJA for 0 h, 6 h and 24 h. The color change from blue to red means the expression level of genes increased gradually. ISPH, 4-hydroxy-3-methylbut-2-en-1-yl diphosphate reductase; transcription factor MYC2; GGPPS (GGPP, GGR), geranylgeranyl diphosphate synthase; OPR (DBR), 12-oxophytodienoic acid reductase; PLA2G (SDP), secretory phospholipase A2; LOX, lipoxygenase; AOC (JMT), allene oxide cyclase; AOS (C74A2), allene oxide synthase; FPPS, farnesyl diphosphate synthase; IDI, isopentenyl-diphosphate delta-isomerase; HMGCR (HMDH), hydroxymethylglutaryl-CoA reductase; DOX, alpha-dioxygenase; ACX (ACOX), acyl-CoA oxidase; JAR1 (GH35), jasmonic acid-amino synthetase.

Jasmonates regulate plant development through a series of hormone-mediated signal transduction, and methyl jasmonate is its main component. α-linolenic acid metabolism plays an important role in jasmonate synthesis, of which allene oxide synthase (AOS), allene oxide cyclase (AOC), lipoxygenase (LOX), and 12-oxophytodienoic acid reductase (OPR) are the essential enzymes (Fig. 4). In this study, α-linolenic acid metabolism was enriched in the KEGG pathway analysis and the majority of the genes functioning in this pathway were up-regulated, with 10 and 16 genes in the 0 h versus 6 h-treated and the 0 h versus 24 h-treated groups, respectively (Fig. 5). In general, it is believed that the application of exogenous JA increases the endogenous jasmonate content. The classical module poses that the JA signaling pathway is activated when plants are stimulated by insects, mechanical injury or exogenous JAs (such as jasmonic acid and MeJA). Subsequently, linolenic acid is converted into its biologically active form, (+)-7-iso-jasmonoyl-L-isoleucine (JA-Ile), which binds to the SCFCOI1 complex receptor that contains the coronatine insensitive 1 (COI1) F-box protein^39–41^. This hormone-receptor interaction causes the degradation of the JAZ repressor proteins by the 26S proteasome, which in turn releases activator proteins such as MYC2 to activate the expression of downstream genes^42^. Here, we found that the jasmonate signaling pathway was enriched in the hormone-mediated signal transduction category in the KEGG pathway analysis for DEGs. In addition, the up-regulation of *AOC, AOS, LOX*, and *OPR* indicated that the α-linolenic acid metabolism pathway was activated by exogenous MeJA in TKS. In this study, we identified numerous genes associated with α-linolenic acid metabolism, such as *JAR1, COI1, JAZ*, and *MYC2*. Among these genes, the expression of *JAR1* and *MYC2* were particularly up-regulated. The pathway showed that MYC2 activated *ORCA3*, which belongs to a family of transcription factors containing the AP2/EREBP domain and is involved in indoleacetic acid and monoterpenoid biosynthesis (Fig. 4).

**Figure 6:**
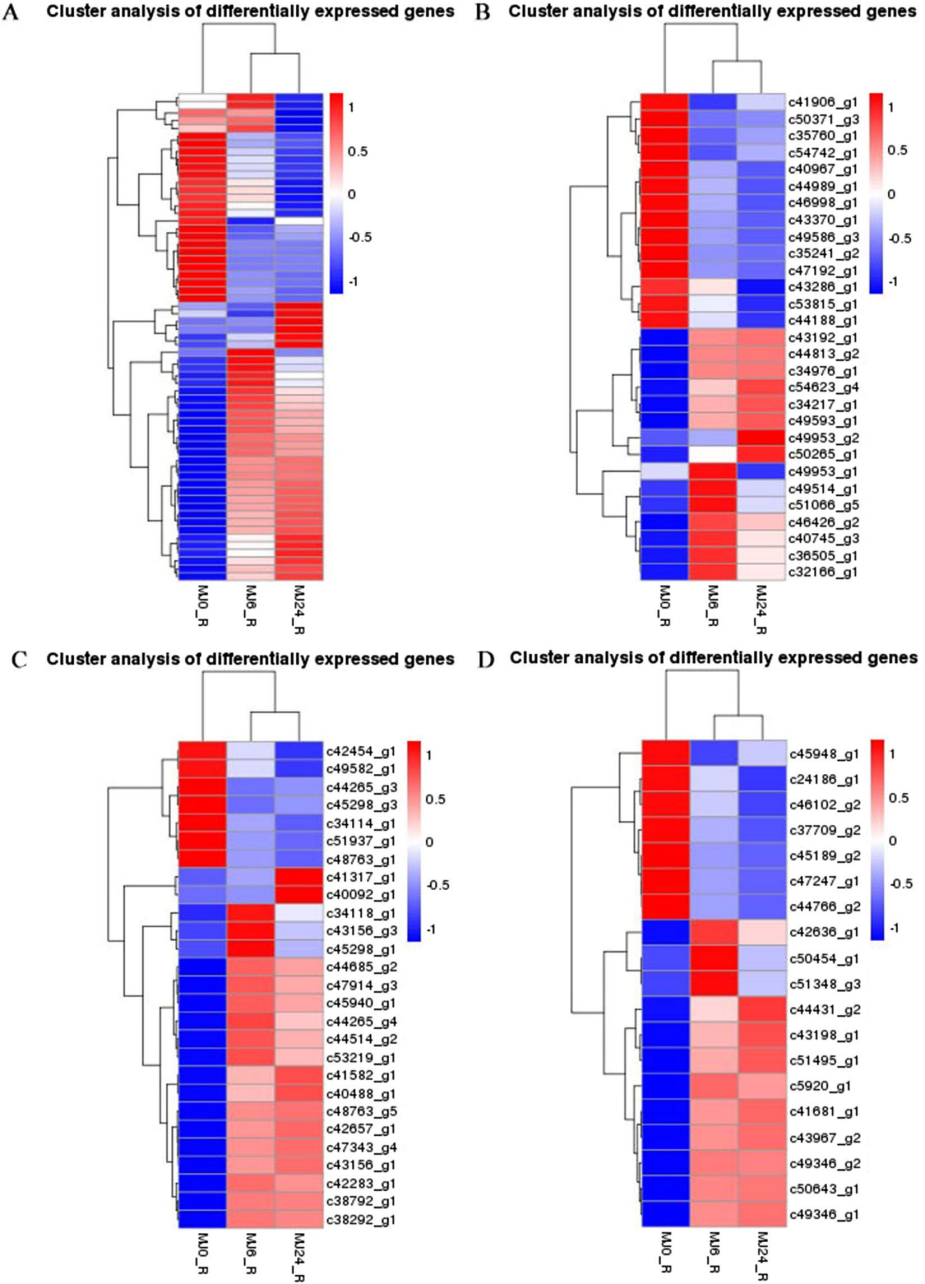
Heat map of differentially expressed genes belong to the transcription factor families AP2/EREBP (**A**), bHLH (**B**), MYB (**C**), and WRKY (**D**). The color change from blue to red indicates that the gene expression increased gradually. MJ0, MJ6, and MJ24 indicate that the lines were treated with MeJA for 0 h, 6 h, and 24 h.

Exogenous stimuli increase the secondary metabolite content in plants through signal transduction, which can be used extensively for various purposes. Vinblastine, is a valid anticancer compound derived from vindoline, which is produced in the Madagascar periwinkle (*Catharanthus roseus*) via the terpenoid indole alkaloids (TIA) pathway. The formation of vindoline requires deacetylvindoline 4-O-acetyltransferase (DAT), whose expression is regulated by JA^43^. In *C. roseus*, CrWRKY, CrMYC1, CrMYC2, CrORCA2, and CrORCA3 are involved in the response to JA^44–46^. Meanwhile, Cr*ORCA3* and Cr*WRKY* overexpression resulted in an increase in the levels of genes like *TDC*, *STR*, and desacetoxyvindoline-4-hydroxylase (*D4H*), which play crucial roles in TIA biosynthesis^47,48^.This suggests that these transcription factors (TFs) may be involved in the regulation of vinblastine biosynthesis in response to JA. Furthermore, several enzymes and their corresponding genes involved in the biosynthesis of nicotine in *Nicotiana* have been identified, for example, arginine decarboxylase (ADC), ornithine decarboxylase (ODC), putrescine N-methyltransferase (PMT), N-methyl-putrescine oxidase (MPO), aspartate oxidase (AO), quinolinate synthase (QS), quinolinic acid phosphoribosyltransferase (QPT), PIP (pinoresinol–lariciresinol reductase, isoflavone reductase, phenylcoumaran benzylic ether reductase) family isoflavone reductase-like protein A622, and berberine bridge enzyme-like protein (BBL)^49^. Studies in *Nicotiana* species revealed that after the degradation of NtJAZs by NtCOI1, NtMYC2 directly regulated the expression of selected nicotine biosynthetic genes like *PMT* by binding to the *PMT* promoter and inducing the expression of *PMT* ^50^. On the other hand, NtMYC2 also regulated the expression of the NIC2 AP2/ERF TFs, following which, NIC2 bound to the GCC-boxes of the *QPRT2* promoters^51^. NtMYC2 regulated nicotine biosynthesis in these two ways in response to JA. Similarly, CrMYC2, which regulated the expression of the AP2/ERF-domain transcription factor ORCA in *Artemisia annua* L species, and AabHLH1 (the MYC2 homolog), which regulated the expression of AaERF1 and AaERF2, also regulated the transcription of artemisinin biosynthetic genes encoding ADS and CYP sesquiterpene oxidase^45,52^. Among the WRKY TFs, WRKY1 trans-activated the promoter of the *ADS* gene by binding to the TTGACC W-box cis-acting elements. The expression of the majority of the artemisinin biosynthetic genes is activated by AaWRKY1^53^. Overexpression of AaERF1 or AaERF2 led to increased accumulation of artemisinin and artemisinic acids^52^. In this study, the expression pattern of TF-encoding genes was analyzed, and 2,538 unigenes of 80 different putative TF families were identified, of which the majority of the unigenes were annotated into 9 TF families, including AP2/EREBP (179), bHLH (96), bZIP (92), C2H2 (154), C3H (111), MYB (172), NAC(83), Orphans (117), and WRKY (78). Our findings suggest that AP2/EREBP, bHLH, MYB, and WRKY play an important function in response to MeJA in TKS. Interestingly, differential expression pattern analysis of these genes showed that the expression pattern of *bHLH, AP2/EREBP, MYB*, and *WRKY* family members in the 6 h-treated lines is consistent with the result of 24 h-treated line (Fig. 6). Most differentially expressed members of the AP2/EREBP family were divided into the ERF and DREB sub-family.

### Natural rubber biosynthesis

**Figure 7:**
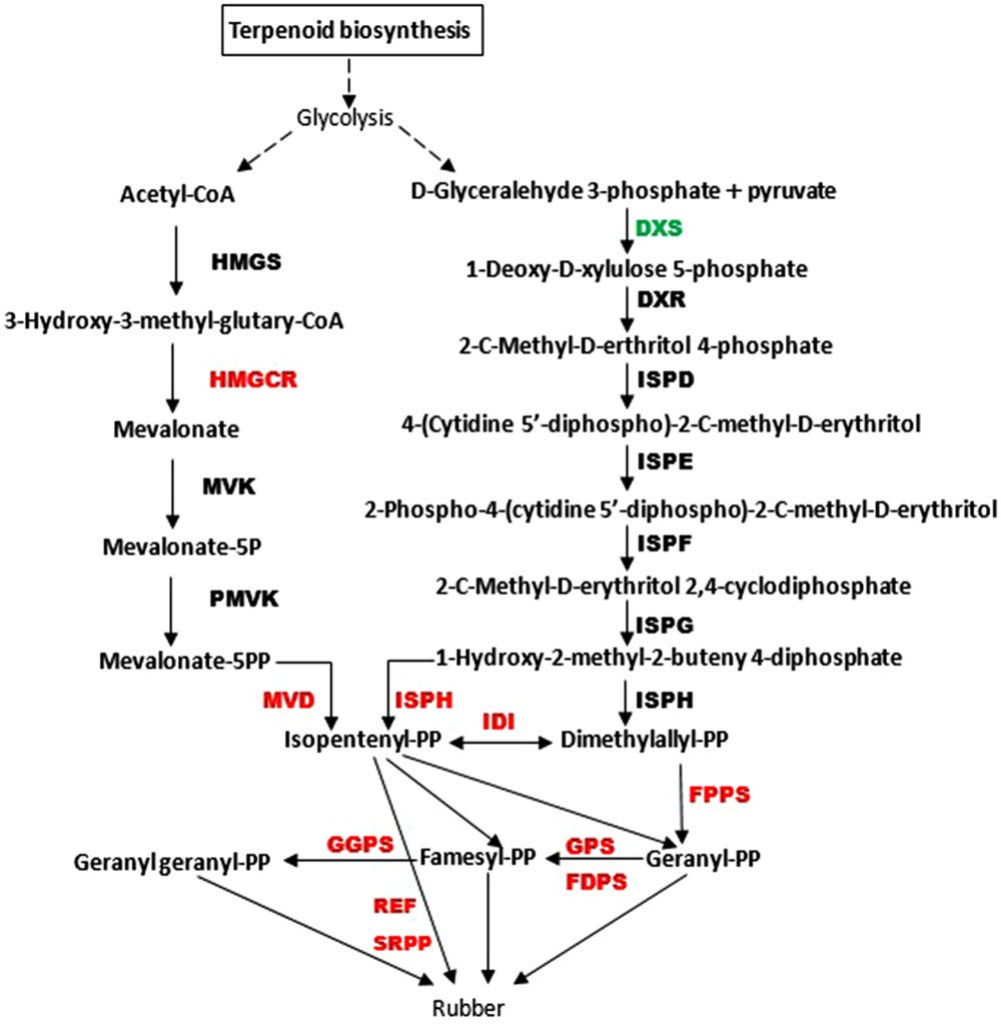
The MVA (left) and MEP (right) pathway for rubber biosynthesis. Red indicates up-regulation of gene expression and green indicates down-regulation of gene expression. The dashed line indicates that the exact process is still unclear. HMGCR, hydroxymethylglutaryl-CoA reductase; MVD, diphosphomevalonate decarboxylase; DXS, 1-deoxy-D-xylulose-5-phosphate synthase; IDI, isopentenyl-diphosphate delta-isomerase; FPPS, farnesyl diphosphate synthase; GGPPS, geranylgeranyl diphosphate synthase; ISPH, 4-hydroxy-3-methylbut-2-en-1-yl diphosphate reductase; SRPP, small rubber particle protein; REF, rubber elongation factor.

Natural rubber is a high molecular weight biopolymer that is composed of cis-isoprene units derived from isopentenyl diphosphate (IPP). The biosynthesis of NR occurs via the mevalonic acid (MVA) and methylerythritol phosphate (MEP) pathways, which differ in the source of IPP. In the MVA pathway, IPP originates from mevalonate that is formed from acetyl-CoA by hydroxyl-methyl-glutaryl-CoA reductase (HMGCR). In contrast, 1-deoxy-D-xylulose-5-phosphate synthase (DXS) catalyzes the reaction between pyruvate and D-glyceraldehyde 3-phosphate to form 1-Deoxy-D-xylulose 5-phosphate, which is subsequently converted to IPP by 4-hydroxy-3-methylbut-2-en-1-yl diphosphate reductase (ISPH) in the MEP pathway. Excess IPP is constantly added to a priming allylic diphosphate (farnesyl diphosphate and/or geranylgeranyl diphosphate) to synthesize cis-isoprene, which is catalyzed by numerous enzymes such as isopentenyl-diphosphate delta-isomerase (IDI), dimethyallyl transferase, farnesyl diphosphate synthase (FPPS), and geranylgeranyl diphosphate synthase (GGPPS) (Fig. 7). Studies on *H. brasiliensis* demonstrated that *HMGCR*, *IDI*, *FPPS*, and *GGPPS* are critical for rubber biosynthesis via the MVA pathway^54–57^. Previous studies revealed that the small rubber particle protein (SRPP) is involved in rubber biosynthesis in TKS^22,24^. The transcriptome analysis showed that the genes essential for NR production, such as *HMGCR, FPPS, GGPPS, IDI*, and *ISPF*, were present in the TKS samples and were expressed highly in the MeJA-treated groups (6 h and 24 h-treated lines) compared to that in the 0 h-treated line (Fig. 5A,B). Transcript c41870_g1, encoded by SRPP, showed high expression in the MeJA-treated samples. However, the expression of *DXS*, an important gene in the MEP pathway, was lower after MeJA treatment. The increased expression of these genes subsequently induced NR accumulation, suggesting that MeJA could stimulate secondary metabolism via the MVA pathways and enhance the NR content of TKS.

### qPCR validation of differential gene expression

**Figure 8:**
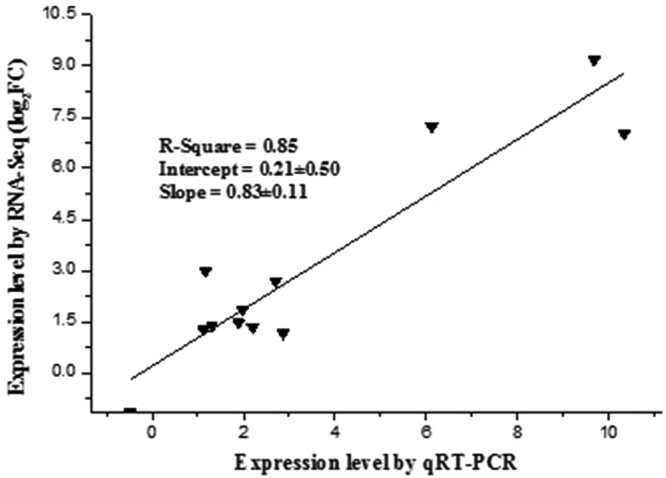
Correlation analysis of the expression of the 12 selected genes by qPCR assay and RNA-seq. The qPCR values for each gene are represented as means ± standard deviation (SD) of three biological replicates. The selected genes were AOS, hydroperoxide dehydratase; AOC, allene oxide cyclase; LOX, lipoxygenase; OPR, 12-oxophytodienoic acid reductase; HMGCR, hydroxymethylglutaryl-CoA reductase; DXS, 1-deoxy-D-xylulose-5-phosphate synthase; IDI, isopentenyl-diphosphate delta-isomerase; FPPS, farnesyl diphosphate synthase; GGPPS, geranylgeranyl diphosphate synthase; ISPH, 4-hydroxy-3-methylbut-2-en-1-yl diphosphate reductase; transcription factor MYC2; SRPP, small rubber particle protein.

Twelve genes from the α-linolenic and terpenoid metabolic pathways were selected to validate the results of differential gene expression analysis obtained from RNA-sequencing using quantitative real-time polymerase chain reaction (qPCR). Six seedlings were selected for the qPCR analyses with three biological replicates. qPCR analysis showed that the expression pattern of these 12 genes were similar to that observed by RNA-seq. Statistical analysis also showed positive correlation (r = 0.85) between the qPCR and RNA-seq datasets (Fig. 8).

### Regulation of NR biosynthesis by *ERF* and *DREB*

**Figure 9:**
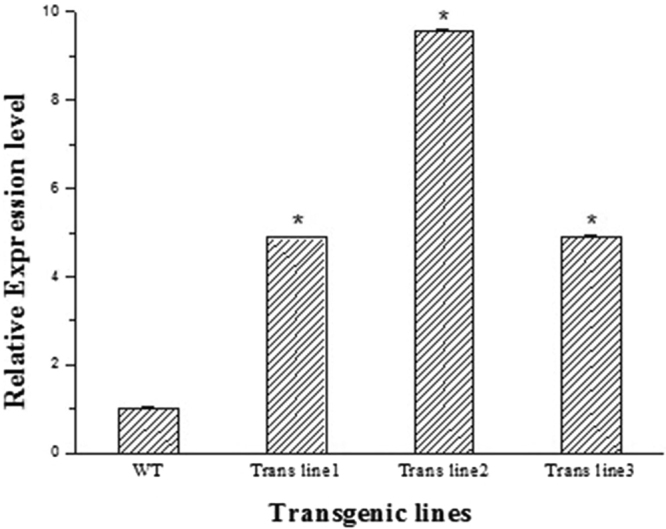
The expression analysis of *HMGCR* by qPCR in the wild type and *DREB* transgenic lines. The values represent the mean value ± SE from three replicates. *Indicates significant differences compared to the WT at *P* < 0.05 (*t*-test).

KEGG pathway enrichment analysis of the DEGs detected the α-linolenic acid metabolism pathway (ko00592), which showed that jasmonic acid could regulate monoterpenoid biosynthesis through *MYC2* and *ORCA3*. ORCA3 is an AP2-domain transcription factor. We obtained genes encoding 179 family members of the AP2-EREBP transcription factor family in our RNA-seq dataset, of which 81 exhibited differential expression; these genes were mainly divided into the ERF and DREB sub-families. Therefore, the representative differentially expressed genes, *ERF* and *DREB*, were overexpressed in TKS to determine if they could regulate rubber biosynthesis. The expression level of *HMGCR, IDI, FPPS, SRPP*, and *GGPPS*, which were important for rubber biosynthesis, was analyzed by qRT-PCR in the *ERF* and *DREB* transgenic TKS. The expression level of HMGCR was 4.88, 9.58, and 4.89 fold higher in 3 randomly selected *DREB* transgenic seedlings compared to that in the wild type (Fig. 9); however, the expression of the other selected genes was similar to that of the wild type. There were no obvious changes in expression in the *ERF* transgenic lines. This suggests that DREB regulates rubber biosynthesis by controlling the expression of *HMGCR* in response to JA. Three HbWRKYs, HbWRKY3, 14, and 55, which were identified from the rubber tree genome and responded to JA treatment were also observed to bind to the *HbSRPP* promoter and activate transcription in yeast^58^.

## Conclusion

In this study, we sequenced transcriptomes from a developmental stage of TKS to understand the mechanism of TKS response to JA. KEGG analysis showed that JA regulates majority of the genes involved in plant secondary metabolism, namely, biosynthesis of JA and α-linolenic acid metabolism. We found an enrichment of α-linolenic acid metabolism in the KEGG pathway, which further regulated terpenoid metabolism through a set of TFs. Among these, the JA-related TFs such as AP2/ERRBP, WRKY, bHLH, and MYB were annotated and the expression pattern of the majority of the members of MYB, AP2/EREBP, and WRKY family in the 6 h-treated line was coincident with that from the 24 h-treated line. The expression of several candidate genes involved in rubber biosynthesis is increased. These results, combined with the data from the transcript assay, suggest that JA regulates rubber biosynthesis through α-linolenic acid metabolism. Further studies to investigate the interaction between TFs with function genes are underway. In addition, the transcriptome data obtained in this study is a valuable information resource for further identification of genes related to rubber biosynthesis.

## Materials and Methods

### Plant growth and treatment

Wild TKS seeds were harvested from Shi He Zi of Xin-Jiang province, and the sterile seeds were then used for tissue culture plantlets. Tissue culture plantlets of wild TKS from Xin-Jiang were grown at a constant temperature of 22 °C in a growth room under a 16 h light/8 h dark photoperiod. Callus cultures were grown on MS (Murashige and Skoog) medium with 30 g L^−1^ sucrose, 30 g L^−1^ agar, 1.0 mg L^−1^, 6-BA (6-Benzylaminopurine), and 0.1 mg L^−1^ NAA (1-naphthylacetic acid) in a culture dish incubator for 15 days. Then, the differentiated callus cultures were transformed into 1/2 MS medium with 30 g L^−1^ sucrose, 30 L^−1^ agar, 0.5 mg L^−1^ NAA. Wild seedlings with well-developed roots were then transplanted into pots containing peat and perlite to maintain the same growth condition. MeJA treatment was performed when the wild seedlings were three-months-old. Nine seedlings of the same growth period and similar height were randomly chosen to be exposed to 0.8 mmol/L MeJA (MeJA was dissolved in absolute ethanol before dilution to the required concentrations with distilled water) for 6 and 24 h, respectively. The control lines were treated for 0 h. The primary and lateral roots were harvested from both the control and experimental populations (three biological replicates were performed). The collected samples were immediately frozen in liquid nitrogen and then stored at −80°C for RNA extraction.

### Total RNA extraction, library preparation and *de novo* sequencing

Total RNA was extracted using the EasyPure plant RNA Kit (Transgen Biotech, China) following the manufacturer’s instructions. RNA quality was monitored by electrophoresis on 1% (w/v) agarose gel, the purity was assessed using the NanoPhotometer® spectrophotometer (Implemen, CA, USA), and the concentration and integrity were assessed using the Qubit® RNA assay kit in the Qubit® 2.0 fluorometer (Life Technologies, CA, USA) and the RNA Nano 6000 assay kit of the Agilent Bioanalyzer 2100 system (Agilent Technologies, CA, USA). mRNA was fragmented after purification from the total RNA to construct sequencing libraries using the NEBNext® Ultra™ RNA library preparation kit for Illumina® (NEB, USA) following the manufacturer’s recommendations, and index codes were added to attribute unique barcodes to each sample. First strand cDNA was synthesized using random hexamer primers and M-MuLV reverse transcriptase (NEB, USA). Second strand cDNA synthesis was subsequently performed using DNA polymerase I and RNase H (NEB, USA). Remaining overhangs were converted into blunt ends via the exonuclease/polymerase activities (NEB, USA). After adenylation of the 3′ ends of the DNA fragments, NEBNext adaptor with hairpin loop structure were ligated. The library fragments were purified with AMPure XP system (Beckman Coulter, Beverly, USA) to select cDNA fragments preferentially 150–200 bp in length. Finally, cDNA libraries were generated and the quality was assessed on the Agilent Bioanalyzer 2100 system. The clustering of the index-coded samples was performed on a cBot cluster generation system using TruSeq PE Cluster Kit v3-cBot-HS (Illumia) according to the manufacturer’s instructions. The library preparations were sequenced on an Illumina Hiseq. 4000 platform and paired-end reads were generated.

### *De novo* transcriptome assembly and functional annotation

Raw data (raw reads) were first processed through in-house perl scripts. In this step, clean data (clean reads) were obtained by removing reads containing adapter sequences, poly-N, and low quality reads from raw data (the number of reads with quality value Qphred ≤ 20 was more than 50%). Transcriptome assembly was accomplished based on the clean paired-end reads using Trinity^59^ with min_kmer_cov set to 2 by default and all other parameters also set to default. The de novo transcriptome served as the reference transcriptome, and the longest transcript was selected as the unigene to represent one gene. Transcriptome annotation was performed using transcript BlastX alignmemt in seven databases such as NR (NCBI non-redundant protein sequences, e-value = 1e^−5^), NT (NCBI nucleotide sequences, e-value = 1e^−5^), Pfam (e-value = 0.01), KOG (e-value = 1e^−3^), Swiss-Prot (a manually annotated and reviewed protein sequence database, e-value = 1e^−5^), and KEGG (e-value = 1e^−10^). The GO annotations were based on the results of NR and Pfam using the Blast2GO v2.5^60^ with e-value = 1e-6. Transcription factors were identified and classified into families using the iTAK pipeline (http://bioinfo.bti.cornell.edu/tool/itak).

### Differential expression analysis

Clean data were mapped back onto the assembled transcriptome and the read count for each gene was obtained from the mapping results. Gene expression levels were estimated by RSEM^61^ for each sample. Differential expression analysis of two groups was performed based on the resulting P values that were adjusted using the Benjamini and Hochberg’s approach for controlling the false discovery rate. Here, genes with an adjusted P-value (adj. P) < 0.05 (found by DESeq) were assigned as differentially expressed. GO enrichment analysis of the differentially expressed genes (DEGs) was implemented using the GOseq.^62^. We used the KOBAS^63^ software to test the statistical enrichment of differential expression in KEGG pathways, and false discovery rate (FDR) ≤ 0.05 was used to select markedly enriched pathways.

### Real-time RT-PCR analysis

Primers of each selected transcript-derived fragment (TDFs) sequence were designed using Primer Premier 6.0 software (USA) (Supplementary Table S1). Quantitative real-time PCR was performed on the Light Cycler 480 system (Roche Diagnostics, Germany) using the Light Cycler 480 SYBR Green master mix (Roche Diagnostics). The PCR components consisted of 500 ng single stranded cDNA, master mix 10 μL, primer 2 μL (forward primer 1 μL, reverse primer 1 μL), and sterile water in a total volume of 20 μL. The amplification program was set for initial denaturation at 95 °C for 5 min, followed by 40 cycles of denaturation at 95 °C for 30 s, annealing at 60 °C for 30 s, extension at 72 °C for 1 min, and a final extension at 72 °C for 10 min. Relative expression was calculated by the 2^−ΔΔCt^ method using the TKS GAPDH as a constitutive control. Analyses were performed with three biological replicates.

### Overexpression of Tk*ERF* and Tk*DREB*

The representative differentially expressed genes, *ERF* and *DREB*, were selected for the overexpression assay. Tk*ERF* (KY436387) and Tk*DREB* (KY436388) were cloned from the respective cDNAs and sequenced. Thereafter, these two genes were transferred to the expression plasmid pCAMBIA2300 containing the CaMV35S promoter and co-transfected in wild TKS by *Agrobacterium tumefaciens* GV3101 (Supplementary Fig. S8).

## Electronic supplementary material

supplementary information

## References

[1] International Rubber Study Group. The Rubber Statistical Bulletin. Singapore, **6**, 62, May/June (2008).

[2] IRSG Rubber Statistical Bulletin. **69**, 10–12, April–June (2015).

[3] Lieberei R (2007). South American leaf blight of the rubber tree (Hevea spp.): new steps in plant domestication using physiological features and molecular markers. Annals of botany..

[4] Barres B (2012). Understanding the recent colonization history of a plant pathogenic fungus using population genetic tools and Approximate Bayesian Computation. Heredity..

[5] Chen SC (2002). Association of decreased expression of a Myb transcription factor with the TPD (tapping panel dryness) syndrome in *Hevea brasiliensis*. Plant Mol Biol..

[6] Li, D.-J. *et al*. Identification and characterization of genes associated with tapping panel dryness from *Hevea brasiliensis* latex using suppression subtractive hybridization. *BMC Plant Biol*. 10.1186/1471-2229-10-140 (2010). 10.1186/1471-2229-10-140 PMC3095288 20618931

[7] Rivano F (2013). Breeding *Hevea brasiliensis* for yield, growth and SALB resistance for high disease environments. Industrial Crops and Products..

[8] Pirrello, J. *et al*. Transcriptional and post-transcriptional regulation of the jasmonate signalling pathway in response to abiotic and harvesting stress in *Hevea brasiliensis*. *BMC Plant Biology*. 10.1186/s12870-014-0341-0 (2014). 10.1186/s12870-014-0341-0 PMC4274682 25443311

[9] Buranov, A. U., Elmuradov, B. J., Shakhidoyatov, K. M., Anderson, F. C. & Lawrence J. P. Rubber-bearing plants of Central Asia. Proceedings of 2005 Annual Meeting of American Association for the Advancement of Industrial Crops: International Conference on Industrial Crops and Rural Development, Murcia, Spain, Sept 17–21; AAAIC: Ames, IA, 639–647 (2005).

[10] Whaley, W. G. & Bowen, J. S. Russian dandelion (*Koksaghyz*). An emergency source of natural rubber. *Misc Publ US Dept Agric*. **618** (1947)

[11] Buranov AU, Elmuradov BJ (2010). Extraction and Characterization of Latex and Natural Rubber from Rubber-Bearing Plants. J Agric Food Chem..

[12] Arias, M. *et al*. First genetic linkage map of *Taraxacum koksaghyz Rodin* based on AFLP, SSR, COS and EST-SSR markers. *Scientific Reports*. 10.1038/srep31031 (2016). 10.1038/srep31031 PMC4973268 27488242

[13] Mooibroek H, Cornish K (2000). Alternative sources of natural rubber. Appl Microbiol Biotechnol..

[14] Beilen JBvan, Poirier Y (2007). Guayule and russian dandelion as alternative sources of natural rubber. Crit. Rev. Biotechnol..

[15] Poulter CD, Rilling HC (1976). Prenyltransferase - mechanism of reaction. Biochemistry..

[16] Poulter CD, Rilling HC (1978). The prenyl transfer-reaction. Enzymatic and mechanistic studies of 1′-4 coupling reaction in the terpene biosynthetic-pathway. Acc Chem Res..

[17] Schmidt, T. *et al*. Characterization of rubber particles and rubber chain elongation in *Taraxacum koksaghyz*. *BMC Biochem*. 10.1186/1471-2091-11-11 (2010). 10.1186/1471-2091-11-11 PMC2836272 20170509

[18] Wahler D (2012). Proteomic analysis of latex from the rubber-producing plant *Taraxacum brevicorniculatum*. Proteomics..

[19] Munt O (2012). Fertilizer and planting strategies to increase biomass and improve root morphology in the natural rubber producer *Taraxacum brevicorniculatum*. Industrial Crops and Products..

[20] Bandurski RS, Teas HJ (1957). Rubber biosynthesis in latex of *Hevea brasiliensis*. Plant Physiol.

[21] Hepper CM, Audley BG (1969). The biosynthesis of rubber from beta-hydroxy-beta-methylgluarylcoenzyme A in *Hevea brasiliensis* latex. Biochem J..

[22] Collins-Silva J (2012). Altered levels of the *Taraxacum koksaghyz* (Russian dandelion) small rubber particle protein, TkSRPP3, result in qualitative and quantitative changes in rubber metabolism. Phytochemistry..

[23] Tata SK (2012). Laticifer tissue-specific activation of the Hevea SRPP promoter in *Taraxacum brevicorniculatum* and its regulation by light, tapping and cold stress. Industrial Crops and Products..

[24] Hillebrand, A. *et al*. Down-regulation of small rubber particle protein expression affects integrity of rubber particles and rubber content in *Taraxacum brevicorniculatum*. *PLoS One*. **7**, 10.1371/journal (2012). 10.1371/journal.pone.0041874 PMC3402443 22911861

[25] De Smet I, Zhang H, Inze D, Beeckman T (2006). A novel role for abscisic acid emerges from underground. Trends Plant Sci..

[26] Finkelstein R, Reeves W, Ariizumi T, Steber C (2008). Molecular aspects of seed dormancy. Annu Rev Plant Biol..

[27] Zhu JK (2002). Salt and drought stress signal transduction in plants. Annu Rev Plant Biol..

[28] Wasternack C, Hause B (2013). Jasmonates: biosynthesis, perception, signal transduction and action in plant stress response, growth and development: an update to the 2007 review in Annals of Botany. Ann Bot..

[29] De Geyter N, Gholami A, Goormachtig S, Goossens A (2012). Transcriptional machineries in jasmonate-elicited plant secondary metabolism. Trends Plant Sci..

[30] Lackmana, P. *et al*. Jasmonate signaling involves the abscisic acid receptor PYL4 to regulate metabolic reprogramming in *Arabidopsis* and *tobacco*. *PNAS*. 10.1073/pnas (2011). 10.1073/pnas.1103010108 PMC3078376 21436041

[31] Fahad, S. *et al*. Exogenously Applied Plant Growth Regulators Enhance the Morpho-Physiological Growth and Yield of *Rice* under High Temperature. *Front Plant Science*. 10.3389/fpls (2016). 10.3389/fpls.2016.01250 PMC5003834 27625658

[32] Coupe M, Lambert C, Primot L, Auzac J (1977). Kinetic action of 2-chloroethyl- phosphonic acid (ethephon) on the latex polysomes of *Hevea brasiliensis*. Phytochemistry..

[33] Stenzel I (2008). The AOC promoter of *tomato* is regulated by developmental and environmental stimuli. Phytochemistry..

[34] Ee SF (2013). Transcriptome profiling of genes induced by salicylic acid and methyl jasmonate in *Polygonum minus*. Mol Biol Rep..

[35] Sun Y (2013). Discovery of WRKY transcription factors through transcriptome analysis and characterization of a novel methyl jasmonateinducible PqWRKY1 gene from *Panax quinquefolius*. Plant Cell Tiss Organ Cult..

[36] Kim OT (2014). Analysis of expressed sequence tags from *Centella asiatica (L.)* Urban hairy roots elicited by methyl jasmonate to discover genes related to cytochrome P450s and glucosyltransferases. Plant Biotechnol Rep..

[37] Ge Q, Xiao Y, Wang Z (2014). Cloning and Characterization of a MeJA-Responsive Jasmonate ZIM-Domain Gene (SmJAZ1) from *Salvia miltiorrhiza Bunge*. Russian Journal of Plant Physiology..

[38] Liu L (2012). Ethylene independent induction of lycopene biosynthesis in tomato fruits by jasmonates. Journal of Experimental Botany..

[39] Chini A (2007). The JAZ family of repressors is the missing link in jasmonate signalling. Nature..

[40] Fonseca S (2009). (+)-7-iso-jasmonoyl-L-isoleucine is the endogenous bioactive jasmonate. Nature Chemical Biology..

[41] Westfall CS (2012). Structural basis for prereceptor modulation of plant hormones by GH3 proteins. Science..

[42] Pauwels L (2010). NINJA connects the co-repressor TOPLESS to jasmonate signaling. Nature..

[43] Goldhaber-Pasillas GD, Mustafa NR, Verpoorte R (2014). Jasmonic acid effect on the fatty acid and terpenoid indole alkaloid accumulation in cell suspension cultures of *Catharanthus roseus*. Molecules..

[44] Li CY (2013). The ORCA2 transcription factor plays a key role in regulation of the terpenoid indole alkaloid pathway. BMC Plant Biol..

[45] Zhang H (2011). The basic helix-loophelix transcription factor CrMYC2 controls the jasmonate-responsive expression of the ORCA genes that regulate alkaloid biosynthesis in *Catharanthus roseus*. Plant J..

[46] Suttipanta N (2011). The transcription factor CrWRKY1 positively regulates the terpenoid indole alkaloid biosynthesis in *Catharanthus roseus*. Plant Physiol..

[47] Schluttenhofer C, Pattanaik S, Patra B, Yuan L (2014). Analyses of *Catharanthus roseus* and *Arabidopsis thaliana* WRKY transcription factors reveal involvement in jasmonate signaling. BMC Genomics..

[48] Wang CT, Liu H, Gao XS, Zhang HX (2010). Overexpression of G10H and ORCA3 in the hairy roots of *Catharanthus roseus* improves catharanthine production. Plant Cell Rep..

[49] Wang X, Bennetzen JL (2015). Current status and prospects for the study of *Nicotiana* genomics, genetics, and nicotine biosynthesis genes. Mol Genet Genomics..

[50] Shoji, T. & Hashimoto, T. Stress-induced expression of NICOTINE2-locus genes and their homologs encoding ethylene response factor transcription factors in tobacco. *Phytochemistry*. 10.1016/j.phytochem (2014). 10.1016/j.phytochem.2014.05.017 24947337

[51] De Boer K (2011). Apetala2/ethylene response factor and basic helix-loop-helix *tobacco* transcription factors cooperatively mediate jasmonate-elicited nicotine biosynthesis. Plant J..

[52] Yu ZX (2012). The jasmonate-responsive AP2/ERF transcription factors AaERF1 and AaERF2 positively regulate artemisinin biosynthesis in *Artemisia annua*. Mol Plant..

[53] Han J, Wang H, Lundgren A, Brodelius PE (2014). Effects of over expression of AaWRKY1 on artemisinin biosynthesis in transgenic *Artemisia annua* plants. Phytochemistry..

[54] Cornish K (2001). Similarities and differences in rubber biochemistry among plant species. Phytochemistry..

[55] Lau, N. S. *et al*. The rubber tree genome shows expansion of gene family associated with rubber biosynthesis. *Scientific reports*. 10.1038/srep28594 (2016). 10.1038/srep28594 PMC5008842 27339202

[56] Adiwilaga K, Kush A (1996). Cloning and characterization of cDNA farnesyl diphosphate synthase from rubber tree (*Hevea brasiliensis*). Plant Molecular Biology..

[57] Venkatachalam P, Priya P, Jayashree R, Rekha K, Thulaseedharan A (2009). Molecular cloning and characterization of a 3-hydroxy-3-methylglutaryl-coenzyme A reductase 1 (hmgr1) gene from rubber tree *(Hevea brasiliensis Muell. Arg*.): A key gene involved in isoprenoid biosynthesis. Physiol Mol Biol Plants..

[58] Li HL, Guo D, Yang ZP, Tang X, Peng SQ (2014). Genome-wide identification and characterization of WRKY gene family in *Hevea brasiliensis*. Genomics..

[59] Grabherr MG (2011). Full-length transcriptome assembly from RNA-Seq data without a reference genome. Nature Biotechnology..

[60] Götz S (2008). High-throughput functional annotation and data mining with the Blast2GO suite. Nucleic Acids Research..

[61] Li, B. & Dewey, C. RSEM: accurate transcript quantification from RNA-Seq data with or without a reference genome. BMC Bioinformatics. 10.1186/1471-2105-12-323 (2011). 10.1186/1471-2105-12-323 PMC3163565 21816040

[62] Young, M. D. *et al*. Gene ontology analysis for RNA-seq: accounting for selection bias. *Genome Biology*. 10.1186/gb-2010-11-2-r14 (2010). 10.1186/gb-2010-11-2-r14 PMC2872874 20132535

[63] Mao X, Cai T, Olyarchuk JG, Wei L (2005). Automated genome annotation and pathway identification using the KEGG Orthology (KO) as a controlled vocabulary. Bioinformatics..

