# Supplementary material for: De novo Transcriptome Sequencing of MeJA-Induced Taraxacum koksaghyz Rodin to Identify Genes Related to Rubber Formation
Source: Sci Rep. 2017 Nov 16;7:15697. doi: 10.1038/s41598-017-14890-z (PMC5691164; doi:10.1038/s41598-017-14890-z)
Supplement: Supplementary file 1 — supplementary information [file 41598_2017_14890_MOESM1_ESM.pdf]

De novo Transcriptome Sequencing of MeJA-Induced *Taraxacum koksaghyz Rodin* to Identify Genes Related to Rubber Formation

XinWen Cao[1], Jie Yan[1*+], JiLian Lei[1], Jin Li[1], JianBo Zhu[1], HuiYan Zhang[1]

[1] College of Life Science, University of Shi He Zi, Xin Jiang, 832000, China
[+] Co-first authors.
[*] Corresponding author

Authors:

**Name**	**Email**
XinWen Cao	981319376@qq.com
JiLiang Lei	raymond8350@yahoo.com
Jin Li	1530804952@qq.com
JianBo Zhu	274831213@qq.com
HuiYan Zhang	spongebobyz@163.com

***Corresponding author:**
Dr. Jie Yan
Department of Life Science,
College of Life Science of Shi He Zi University
Shi He Zi, Xin Jiang, 832000, China
Phone (or Mobile) No.: +8613677564159
Email: 1653842328 @qq.com

Supplementary Figures:

Supplementary Fig S1. The species classification of the NR annotation result of the transcriptome

Supplementary Fig S2. E-value distribution of NR database annotation of the transcriptome

Supplementary Fig S3. Similarity distribution of NR database annotation of the transcriptome

Supplementary Fig S4. Gene function classification of GO dantabase of the transcriptome from three categories of Biological Process, Cellular Component and Molecular Function. The X-axis showed the concrete terms of three categories and the Y-axis showed the number of genes in different terms.

Supplementary Fig S5. KOG function classification of the transcriptome from 26 terms. The Y-axis showed the percentage genes accounted for.

Supplementary Fig S6. DEGs statistics between 0 h and 6h-treated lines (A) and between 0 h and 24 h-treated lines (B). The color red means genes up-regulated and the green means genes down-regulated and the color blue means the expression level of genes showed no obvious change.

Supplementary Fig S7. Enriced terms of gene ontology for the DEGs between the 0 h and 6 h-treated line (A) and between the 0 h and 24 h-treated line (B). Distribution of differential expressed genes in 0 h vs 6h-treated line and 0 h vs 24h-treated line from the categories of biological process (BP) and molecular function (MF); The color red means up-regulated and the blue means down-regulated terms. The Y-axis showed the number of genes for different terms.

Supplementary Fig S8A. The identification of *ERF* transgenic TKS seedlings by genomic pcr. M means DNA Mark Ⅲ; 1 means the positive control and the 2 means the WT, 3-13 means different transgenic seedlings.

Supplementary Fig S8B. The identification of *DREB* transgenic TKS seedlings by genomic pcr. M means DNA Marker Ⅲ and number 1-8 means different transgenic seedlings, 9 means the positive control.

Supplementary Table:

Supplementary Table S1. Primers of 12 selected genes designed by Primer Premier 5.0 used for qPCR analysis

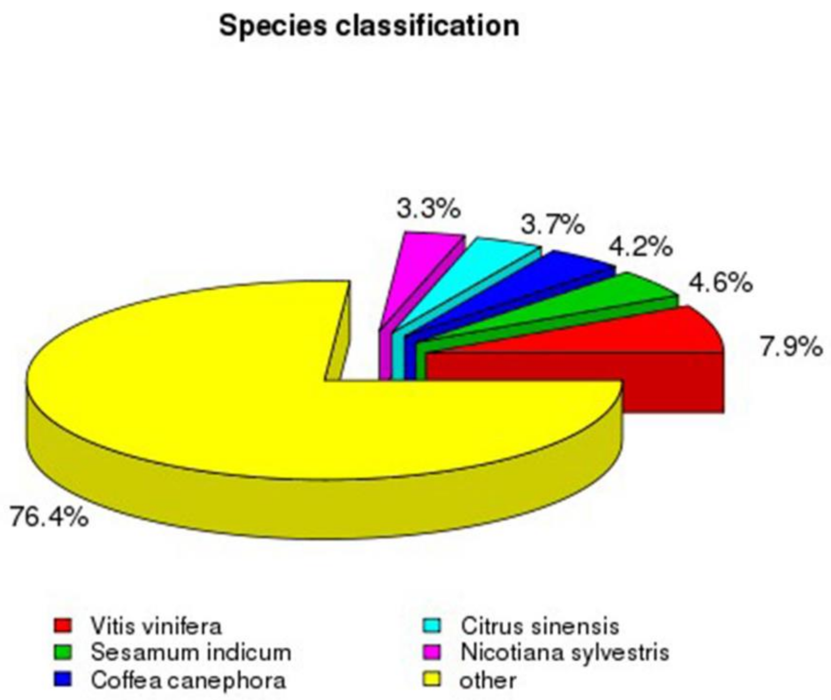

Supplementary Fig S1. The species classification of the NR annotation result of the transcriptome

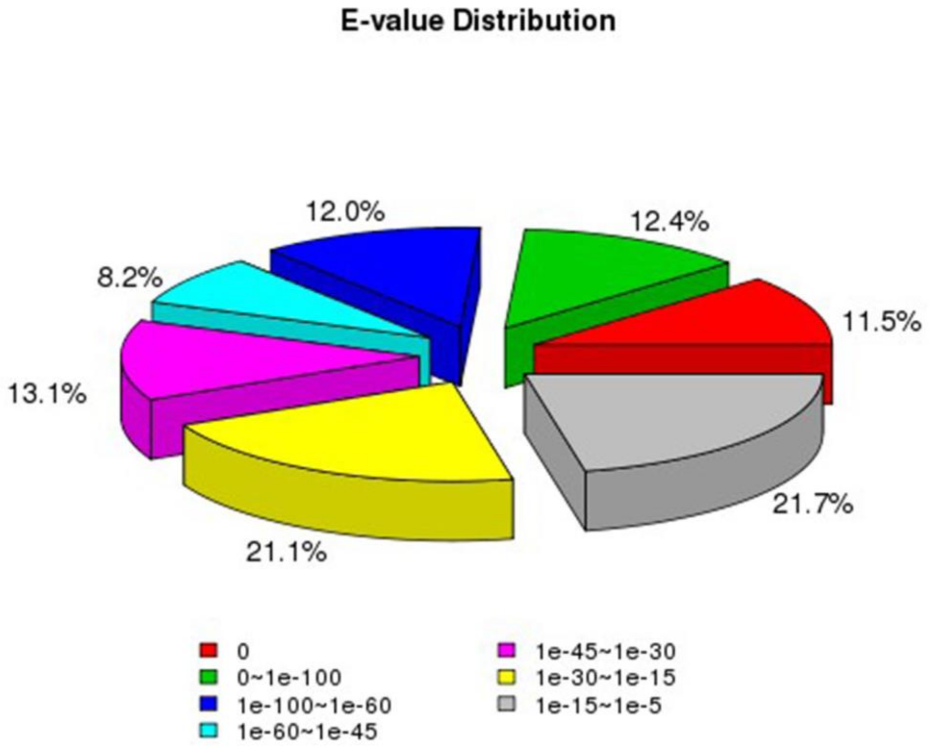

Supplementary Fig S2. E-value distribution of NR database annotation of the transcriptome

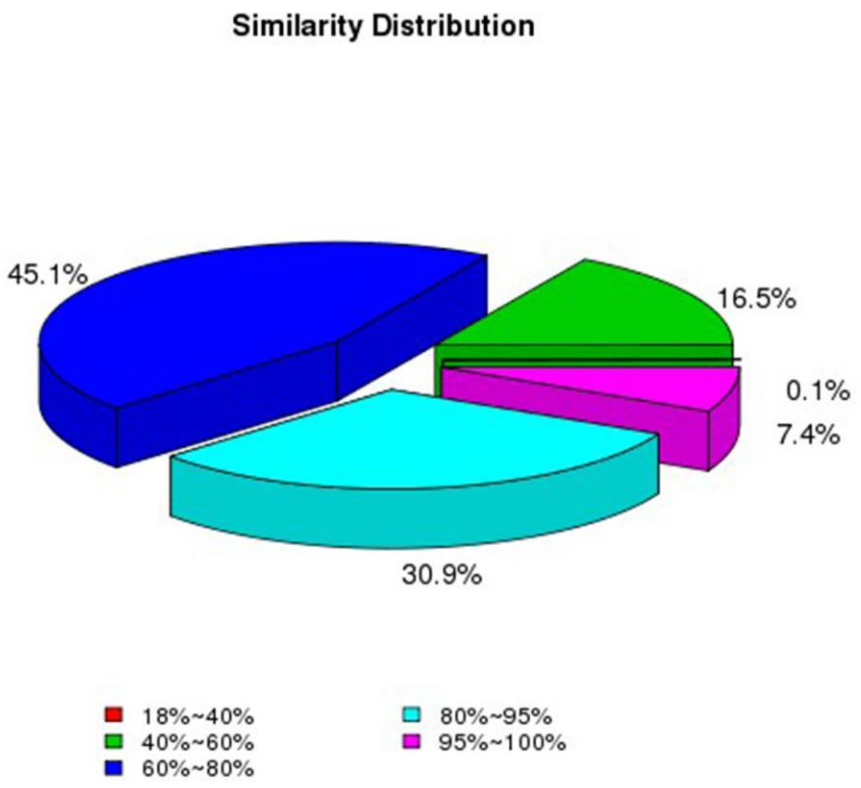

Supplementary Fig S3. Similarity distribution of NR database annotation of the transcriptome

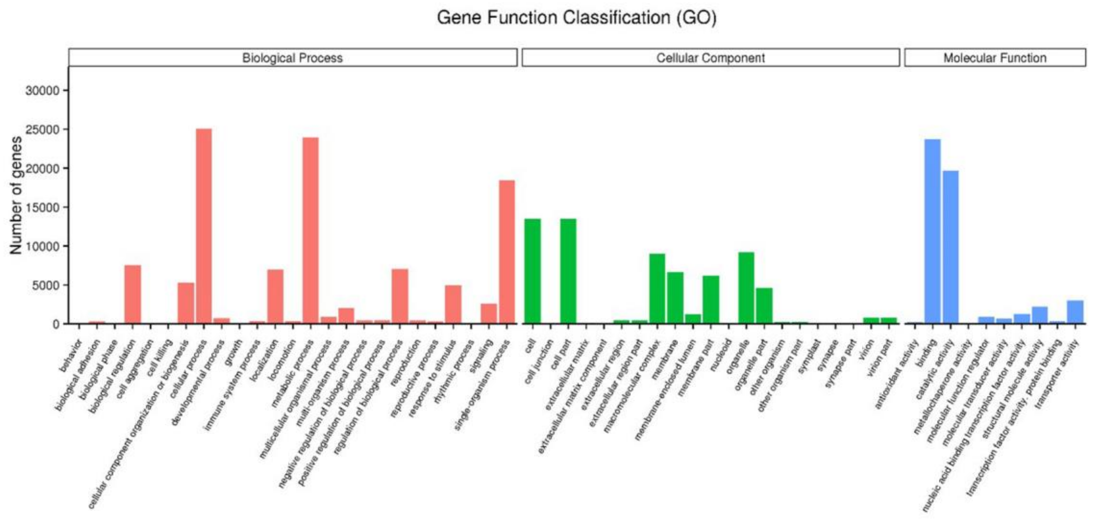

Supplementary Fig S4. Gene function classification of GO dantabase of the transcriptome from three categories of Biological Process, Cellular Component and Molecular Function. The X-axis showed the concrete terms of three categories and the Y-axis showed the number of genes in different terms.

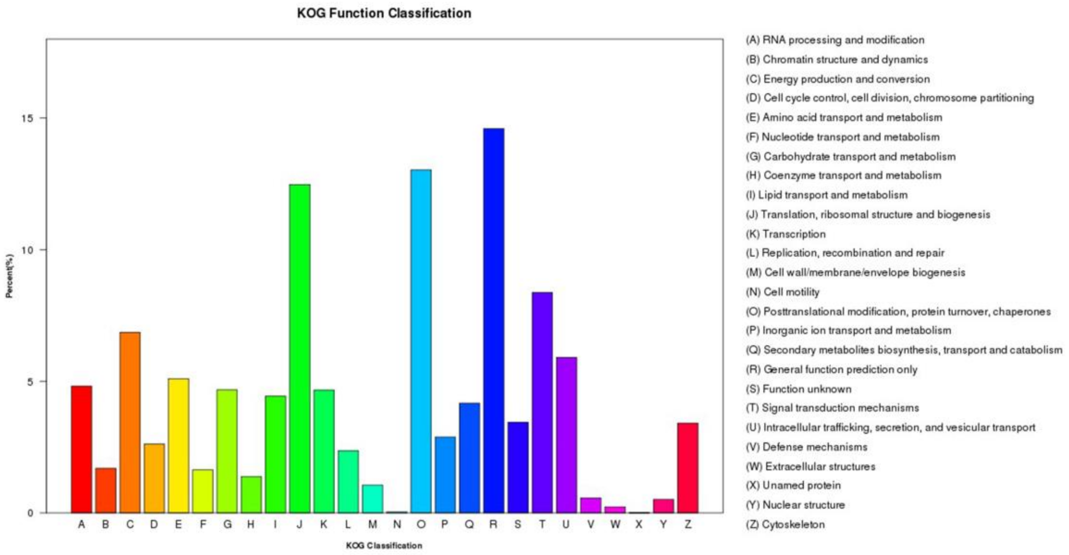

Supplementary Fig S5. KOG function classification of the transcriptome from 26 terms. The Y-axis showed the percentage genes accounted for.

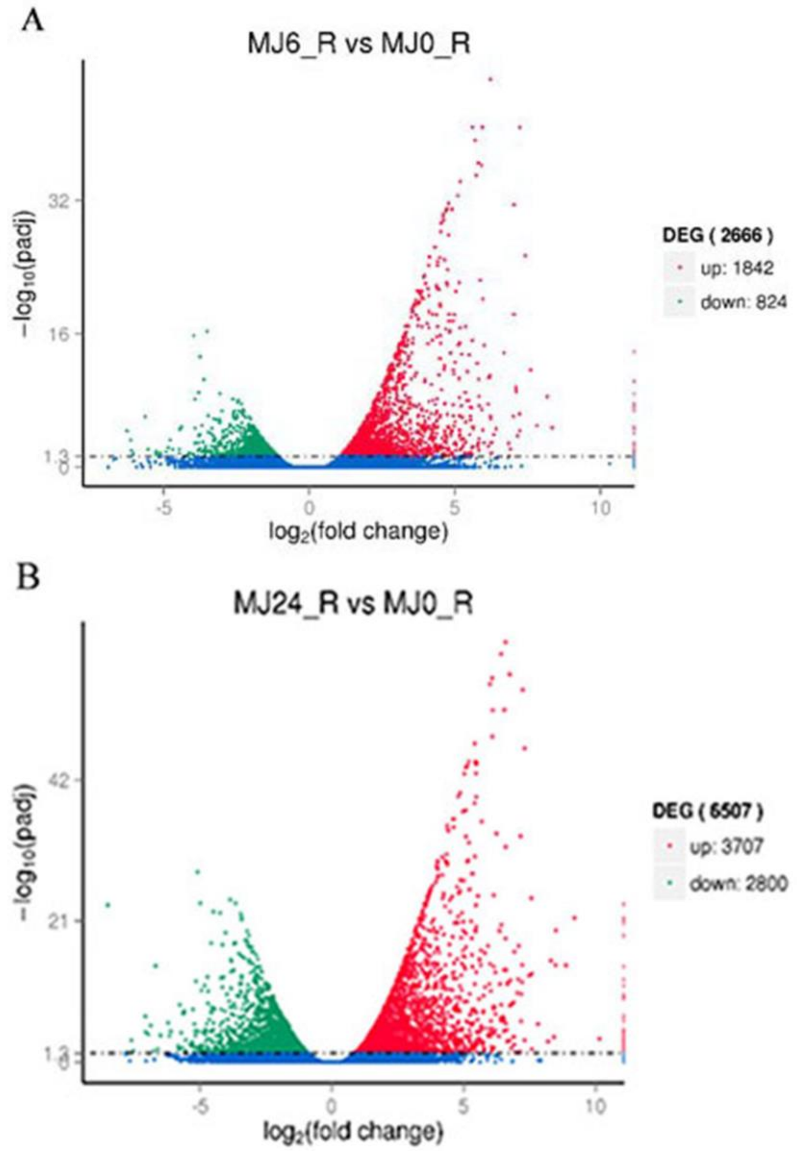

Supplementary Fig S6. DEGs statistics between 0 h and 6h-treated lines (A) and between 0 h and 24 h-treated lines (B). The color red means genes up-regulated and the green means genes down-regulated and the color blue means the expression level of genes showed no obvious change.

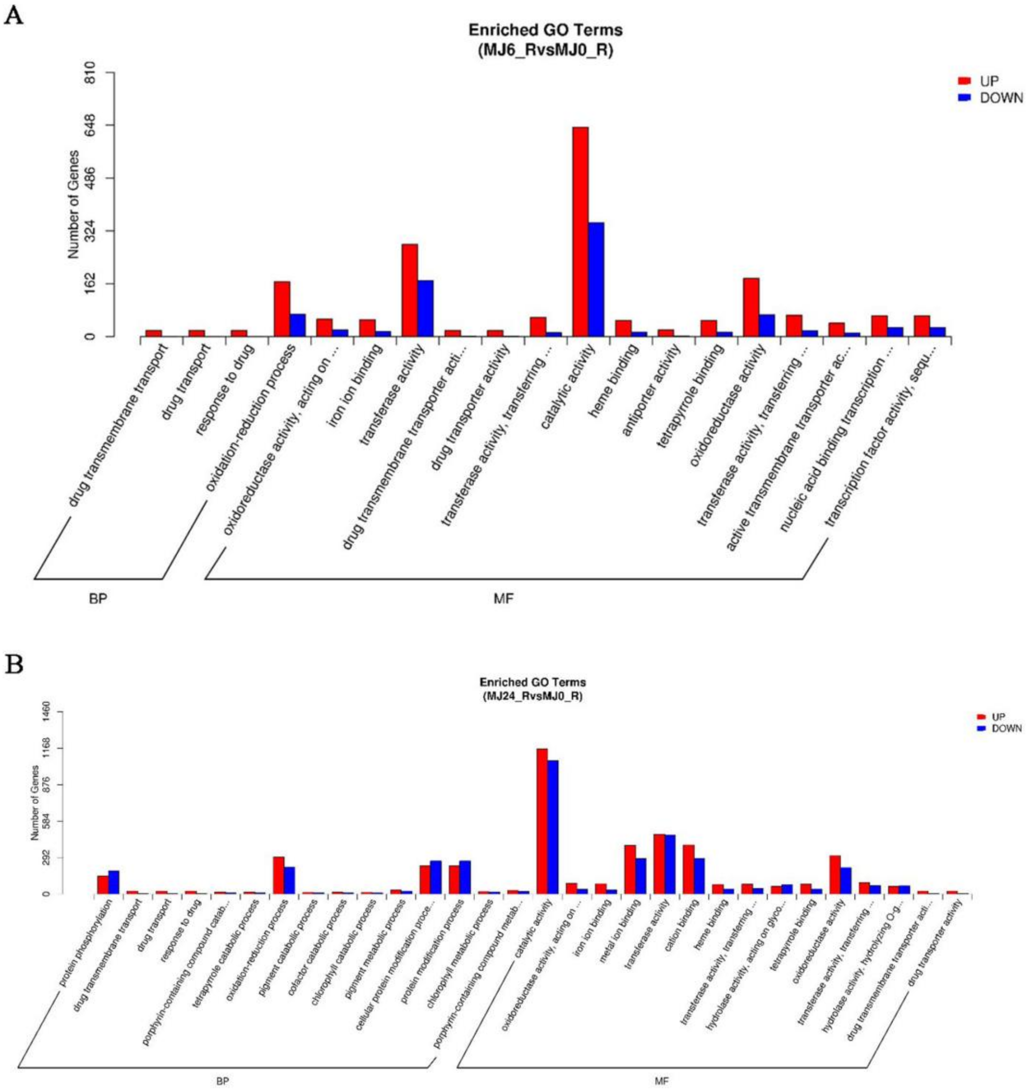

Supplementary Fig S7. Enriced terms of gene ontology for the DEGs between the 0 h and 6 h-treated line (A) and between the 0 h and 24 h-treated line (B). Distribution of differential expressed genes in 0 h vs 6h-treated line and 0 h vs 24h-treated line from the categories of biological process (BP) and molecular function (MF); The color red means up-regulated and the blue means down-regulated terms. The Y-axis showed the number of genes for different terms.

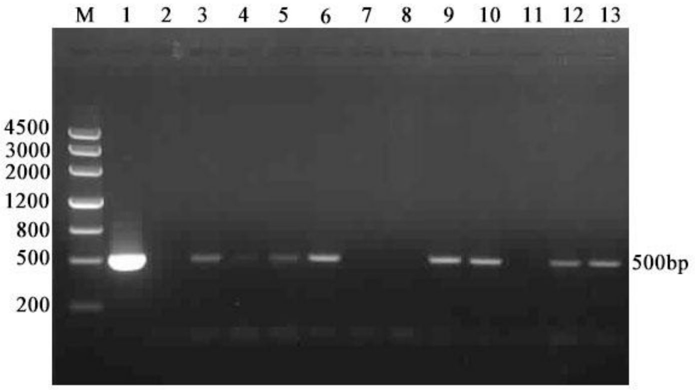

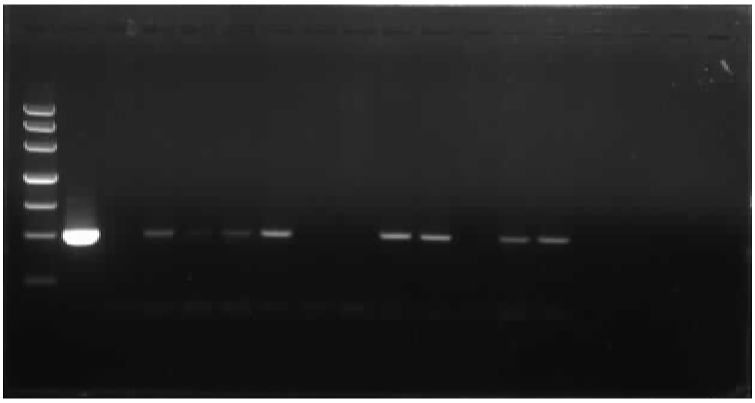

(The original gel image)

Supplementary Fig S8A. The identification of *ERF* transgenic TKS seedlings by genomic pcr. M means DNA Mark Ⅲ; 1 means the positive control and the 2 means the WT, 3-13 means different transgenic seedlings. The gel image acquisition was achieved by Gel Doc XR System (Bio-rad: Intas Gel Hood) and processed with Photoshop (label and clip).

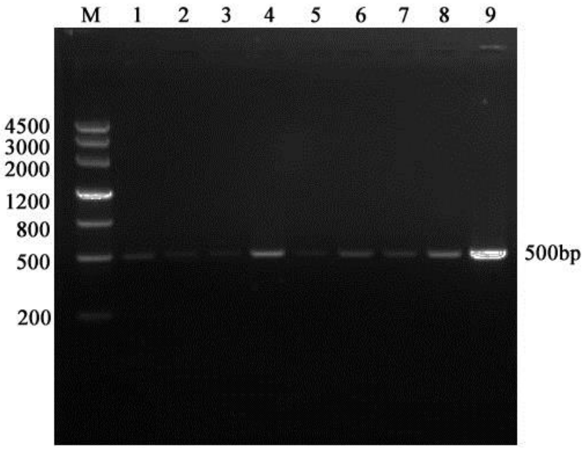

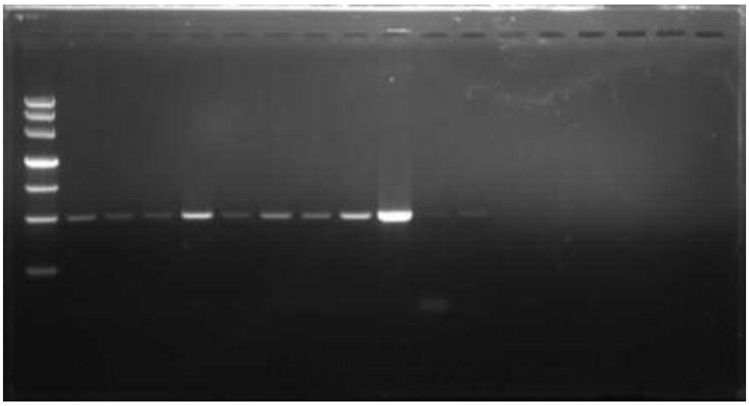

(The original gel image)

Supplementary Fig S8B. The identification of *DREB* transgenic TKS seedlings by Genomic PCR. M means DNA Marker Ⅲ and number 1-8 means different transgenic seedlings, 9 means the positive control. The gel image acquisition was achieved by Gel Doc XR System (Bio-rad: Intas Gel Hood) and processed with Photoshop (label and clip).

Supplementary Table S1. Primers of 12 selected genes designed by Primer Premier 5.0 used for qPCR analysis

Primers used for quantitative PCR analysis

Gene	Unigene ID	Forward Primer	Reverse Promer
AOS	c39329_g1	CTACGGCGAAGGTAAGGTGAC	CTGGAACCCGAATAACATCTCAC
AOC	c44998_g3	ACGGGCAGATAGCAGTCCAG	ACGGAGGAACAGGGGTGACT
LOX	c53508_g1	ATTCTCCGCCAAGCGACCAC	CAACGCCGCCAACACCTA
OPR	c49640_g1	CAGACAAAGTCGGCGTCAGAAT	TCAACTCTGCCACTTCCTCTTCA
MYC2	c46426_g2	GGCGTCGTCATCTGCTTCAA	CCATCTCCCCACCCTAACAC
DXS	c49476_g1	TGCGGGCTACTTGGATTCG	GAACCACTAATCATTCCACGAGC
ISPH	c44729_g5	TTACCTGCTTTCGGTGCTGC	CGGTTGCCACTGTCTCCTCA
HMGR	c50260_g1	TGTCTTCTTCACCGTATTCTTCTCG	CCGAAGAACCCGAGAAGGTAGAT
FPS	c48867_g1	CCTTCGAGTTCGATGACGATTC	TCCGCCTTTAAGCAACTGGTAG
IDI	c43940_g2	CTCTTCCTTCGCTGGTCTTACTCTC	CCCAACGACATTGTCATTTTCATCC
GGPS	c42408_g1	CGGTGACAACTCCAGCAAGATACT	CCTTTCAATCTCCTCGTGGCT
REF/SRPP	c41870_g1	TAATGCCTCCTGCGGTGAAAGAC	AGAGTTTTCCAAGCCGAAGAAGCGT
